# Interactome determination of a Long Noncoding RNA implicated in Embryonic Stem Cell Self-Renewal

**DOI:** 10.1038/s41598-018-34864-z

**Published:** 2018-12-04

**Authors:** Keriayn N. Smith, Joshua Starmer, Terry Magnuson

**Affiliations:** Department of Genetics, University of North Carolina, Chapel Hill, NC 27599 USA

## Abstract

Long noncoding RNAs (lncRNAs) constitute a significant fraction of mammalian transcriptomes and they have emerged as intricate regulators of many biological processes. Their broad capacity to adopt diverse structures facilitates their involvement in the transcriptional, translational and signaling processes that are central to embryonic stem (ES) cell self-renewal and pluripotency. While lncRNAs have been implicated in ES cell maintenance, detailed analyses of those that show significant expression in ES cells is largely absent. Moreover, cooperative molecular relationships that facilitate lncRNA action are poorly understood. Cyrano is a developmentally important lncRNA, and in ES cells, it supports gene expression network maintenance, cell adhesion and cell survival. We have interrogated the interactome of Cyrano to identify protein partners and find that Cyrano is involved in multiple protein networks. We identify a developmentally important cell-signaling hub and find STAT3 as a candidate through which Cyrano can function to reinforce self-renewal of ES cells. Based on commonalities between ES cells and cancer cells, we postulate such functional interactions may support cell proliferation, cell identity and adhesion characteristics in rapidly proliferating cell types. The interactome data will therefore provide a resource for further investigations into interactions that regulate Cyrano or mediate its function.

## Introduction

Long noncoding RNAs (lncRNAs; >200 nt) use multiple mechanisms to function as important regulators of numerous biological processes^1,2^. Intermolecular interactions are central to these regulatory roles^3–6^, yet the protein partners for the vast majority of lncRNAs remain unknown.

Primarily, methods used to interrogate lncRNA-protein interactions are protein-centric, and are limited to known RNA-binding proteins (RBPs)^7–9^. Identifying new RNA-binding proteins is limited because methods are often based on homology to RNA-binding domains which have been previously identified^10^. Thus, unknown RBPs may remain undiscovered. Furthermore, RNA-interactomes are studied using approaches that often favor mRNA interactions, such as oligo(dT) capture-based methods^10^. Lastly, noncoding RNA binding studies are frequently based on *in vitro* studies. While this is informative, these methods only suggest the potential for binding without having a biologically relevant context.

Embryonic stem cells are pluripotent derivatives of the inner cell mass of blastocyst-stage embryos^11–13^. Based on their developmental plasticity and their capacity for unlimited self-renewal, they hold significant potential for use in regenerative medicine, and are important models for dissecting early developmental processes.

While numerous lncRNAs are expressed in ES cells, only a limited number have been functionally implicated in pluripotent cell maintenance through broad screens and/or individual candidate studies^14–17^. These studies show lncRNAs act in transcriptional regulation and post-transcriptional regulatory roles to support self-renewal and pluripotency of ES cells. We propose that the role of lncRNAs in pluripotent stem cell biology requires further study, particularly the intermolecular interactions through which lncRNAs contribute to ES cell maintenance. In this report, we expand the network of the developmentally important lncRNA Cyrano, which we have previously shown supports the characteristics of ES cell self-renewal, to include proteins with which it may function to support ES cell maintenance.

## Results

### Molecular Characteristics of lncRNA Cyrano

Cyrano (1700020I14Rik in mouse, linc-oip5 and Oip5-AS1 in human) is an ~8-9 kb lncRNA with regions of significant conservation among vertebrates^18^. Cyrano is often described as an archetypal lncRNA, yet previous work has demonstrated atypical characteristics, including a highly conserved sequence block that includes a sequence almost perfectly complementary to miR-7^18^. In order to understand how Cyrano functions, we investigated molecular characteristics of the most predominantly expressed mouse and human Cyrano splice variants.

**Figure 1:**
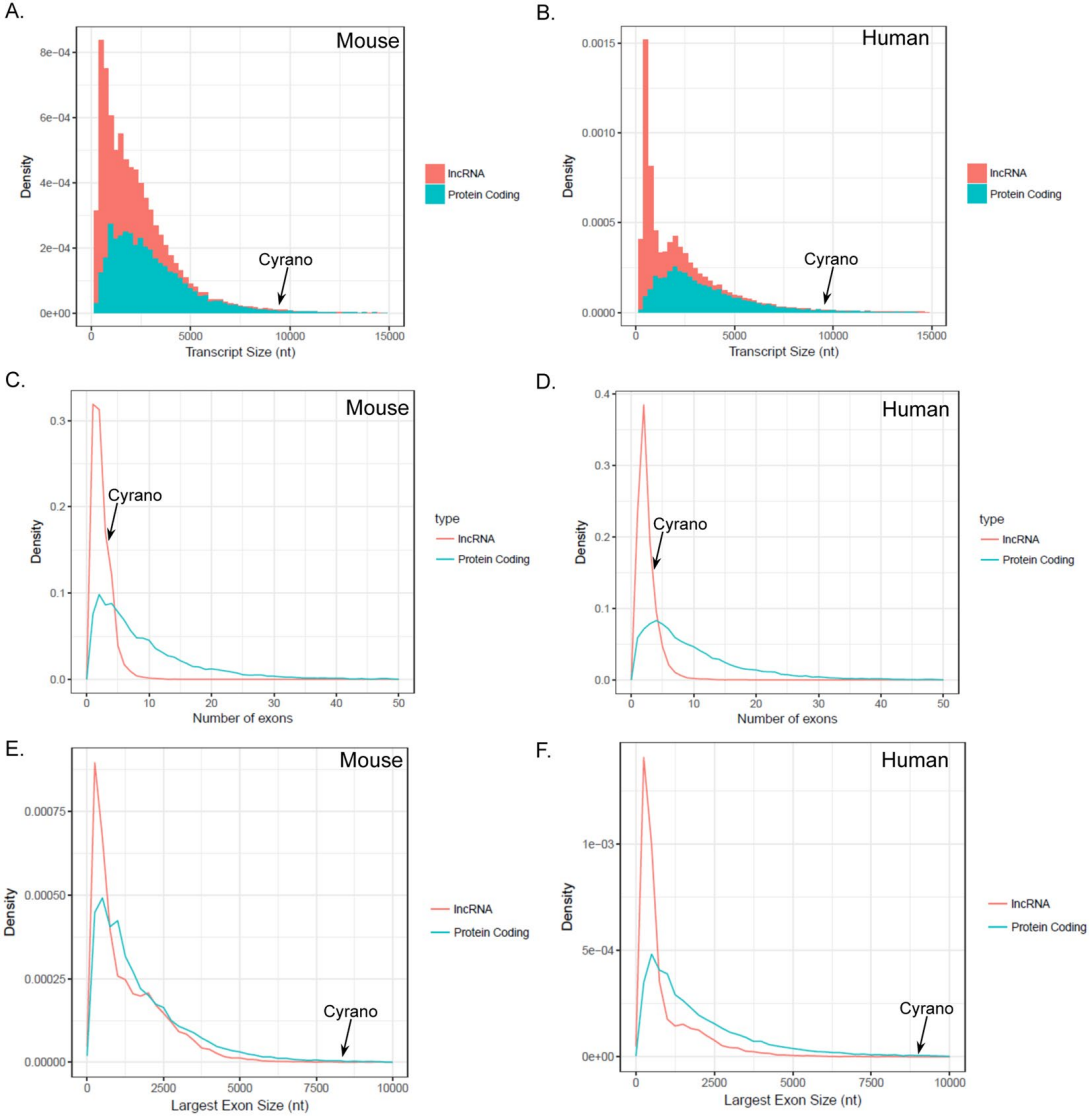
Molecular characterization of the lncRNA Cyrano. (**A**) Comparative analysis of lncRNA and mRNA transcript length (**A**,**B**) of mouse Cyrano (GENCODE v15) and human OIP5-AS1 (GENCODE v27), exon number (**C**,**D**), and longest exon (**E**,**F**).

We assessed Cyrano’s genomic characteristics relative to all lncRNAs and mRNAs and found that Cyrano is among the longest lncRNA transcripts (Fig. 1A,B) in mouse (Cyrano/1700020I14Rik; p-value: 0.0019) and human (OIP5-AS1; p-value: 0.003). The majority of lncRNA genes have few exons, and based on this, skew towards smaller lengths relative to all genes^19–21^. Cyrano’s exon number is not unusual relative to all lncRNAs for mouse (3 exons; p-value: 0.195) and human (4 exons; p: 0.0958) (Fig. 1C,D), indicating that the number of exons is not the primary factor in the transcript length determination. We found that the most significant contributor to the Cyrano transcript length is the size of the largest exon (mouse, p-value: 0.0017; human, p-value: 0.0026), which is approximately 7.9 and 8.5 kb for mouse Cyrano and human OIP5-AS1, respectively.

**Figure 2:**
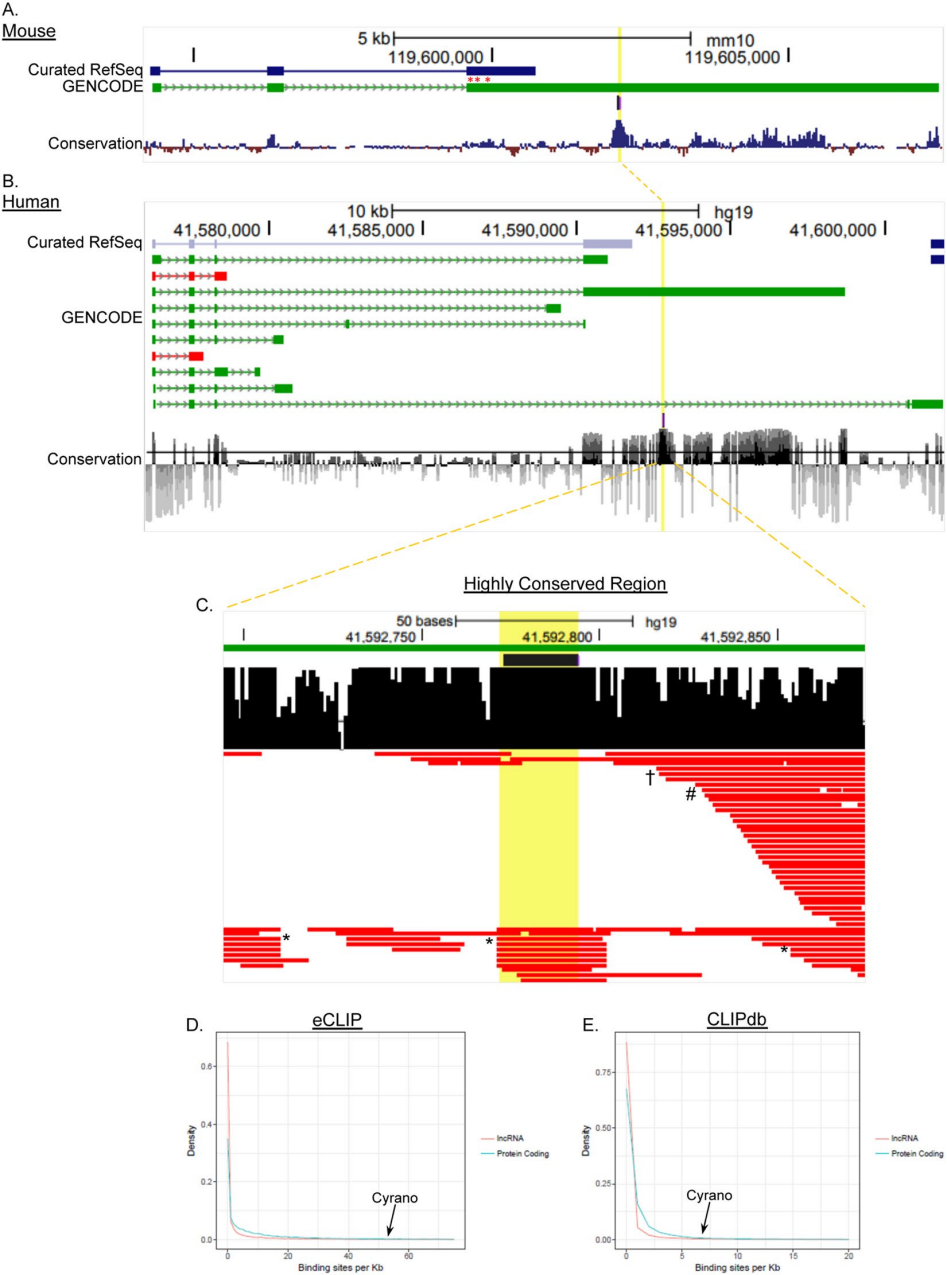
RNA binding protein enrichment for the lncRNA Cyrano. UCSC genome browser shots displaying characteristics of Cyrano including exon annotation, conservation among vertebrates, RNA binding protein sites, and probe locations for mouse Cyrano isolation (red asterisks) (**A**–**C**). The long 3′ terminal exon contains a highly conserved region (**A**,**B**; yellow line, yellow dashed line), that is enriched for RBPs as previously identified in CLIP experiments for OIP5-AS1 (**C**). Data for (**C**) were extracted using POSTAR^22^. Examination of the propensity for RBP binding extends across the entire major transcript (**D**,**E**), relative to other RNAs. Data used are eCLIP (**D**) and CLIPdb (**E**). ^*^ELAVL1, ^†^SND1, ^#^ILF3.

Indeed, the conserved miR-7 interacting site^18^ for both mouse and human Cyrano is located in this long 3′ exon (Fig. 2A,B), which has also been found to mediate interactions with epigenetic regulators such as EZH2^22^. In addition to the demonstrated role for this conserved section in zebrafish^18^, we found that functional interactions mediated through this region are important for Cyrano’s function in ES cells^17^. To investigate the potential of the conserved region within the 3′ exon to mediate intermolecular interactions, we used CLIP data to probe protein interactions in this region of Cyrano^22^. We found that this region has the potential for binding of multiple proteins, possibly in a context-dependent manner (Fig. 2C). This is similar to the broad potential of Cyrano to bind to numerous miRNAs^23^. We next extended the analysis of bound RBPs, as assessed by CLIP, using the full Cyrano transcript. Using both eCLIP and CLIPdb data, we found that Cyrano has the potential to be bound densely by RBPs (Fig. 2D,E). As these are combined analyses from multiple cell types, they suggest context-specificity in Cyrano-RBP binding. However, these data are limited based on the RBPs selected for CLIP.

### Mapping the Cyrano Interactome

**Figure 3:**
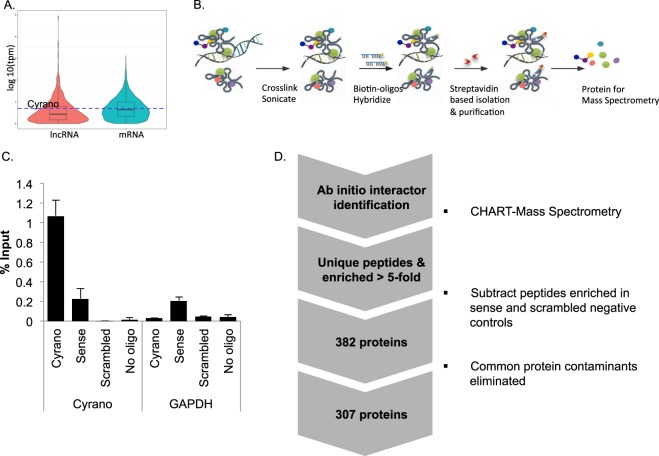
Cyrano is enriched in ES cells where it possesses a complex interactome. (**A**) Comparative transcriptome analysis for Cyrano in ES cells^17^ relative to all lncNRAs. (**B**) The CHART method was used to isolate Cyrano-associated proteins. (**C**) Enrichment of Cyrano RNA using antisense probes in CHART, assessed by qRT-PCR compared to sense and scrambled controls. (**D**) Workflow and proteins identified using CHART-Mass spectrometry with antisense or sense and scrambled controls showing proteins unique to Cyrano and/or enriched 5-fold or greater over sense and scrambled controls. (See also Supplementary Tables S1–3).

We have previously described some functions of Cyrano in ES cells, where its expression is robust relative to other lncRNAs (Fig. 3A), and single molecule assessment revealed that Cyrano is dispersed throughout the nucleus and cytoplasm in mouse and human ES cells^17^. This differs from many other lncRNAs that are nuclear-localized^24^. It is therefore unknown whether Cyrano’s distribution in the cell means that nuclear and cytoplasmic Cyrano pools have distinct cellular functions, whether Cyrano interacts with proteins that shuttle between cellular compartments, or how much its localization depends on its interacting partners. Unbiased identification of interacting proteins using a proteomics approach would provide insight into this, and also identify the protein interactions that mediate Cyrano function or regulation in ES cells.

To facilitate the identification of proteins that interact with Cyrano *in vivo*, we used formaldehyde crosslinking in the affinity pulldown method, Capture Hybridization Analysis of RNA Targets (CHART)^25–27^ to purify Cyrano-protein complexes (Fig. 3B) from whole cell extracts. We were able to enrich for Cyrano RNA using biotinylated antisense probes (Fig. 3C) relative to sense and scrambled control probes.

Candidate interacting proteins were identified using quantitative mass spectrometry (Fig. 3D), which provided a pool of 307 candidate interacting proteins that were unique to the antisense probeset pulldown, combined with those proteins enriched at a threshold of >5-fold in Cyrano pulldown using antisense probes relative to independent sense and scrambled controls (Supplementary Tables S1–S3).

**Figure 4:**
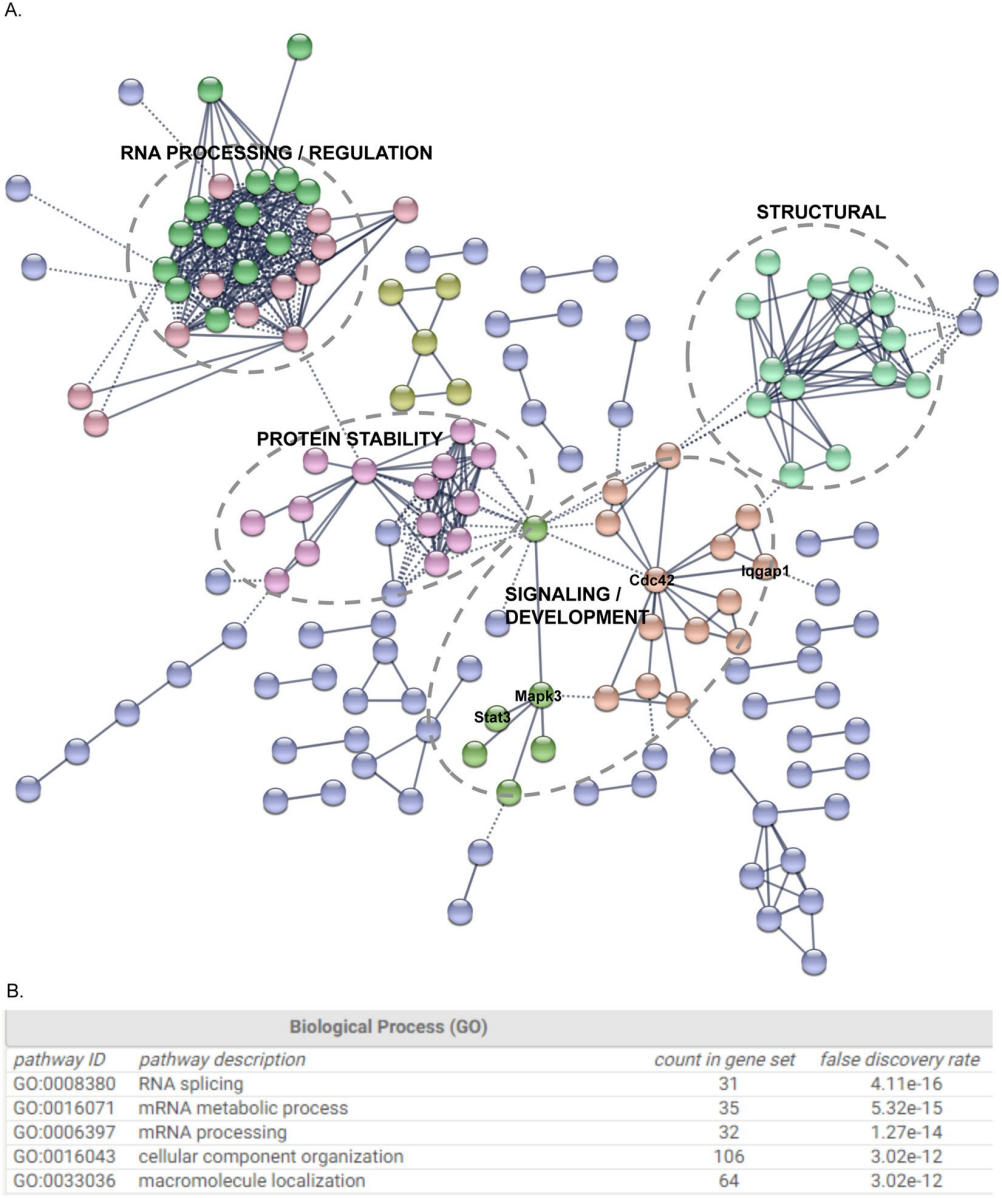
Cyrano-Protein Network in ES cells. (**A**) STRING-generated networks analysis was used to identify major Cyrano-interacting hubs. Protein nodes generate subnetworks, or are unassigned (light violet). The networks are used to annotate candidate functional categories for follow-up, these are highlighted using dashed circles. The identified networks were primarily involved in RNA processing and structural functions (**B**).

Similar to Cyrano, candidate protein interactors (Supplementary Tables S1, 2) were found in both nuclear and cytoplasmic compartments. Our studies identified proteins previously found in CLIP experiments, including ELAVL1, FUS, ILF3, SRSF1, SRSF2, PTBP1 and various HNRNPs (Fig. 2C), suggesting that the candidate proteins contained true partners and were not artefacts of shotgun proteomics. In order to delineate meaningful interactors for further study, we generated protein interacting networks using STRING^28^. These analyses, combined with gene ontology assessments indicated that Cyrano interactors segregated into four primary networks (Fig. 4A,B): (i) an RNA processing/regulatory network; (ii) a network involved in regulating protein stability; (iii) a structural network; and iv) a signaling/developmental network.

### A Cyrano-STAT3 Interaction in Stem Cells

**Figure 5:**
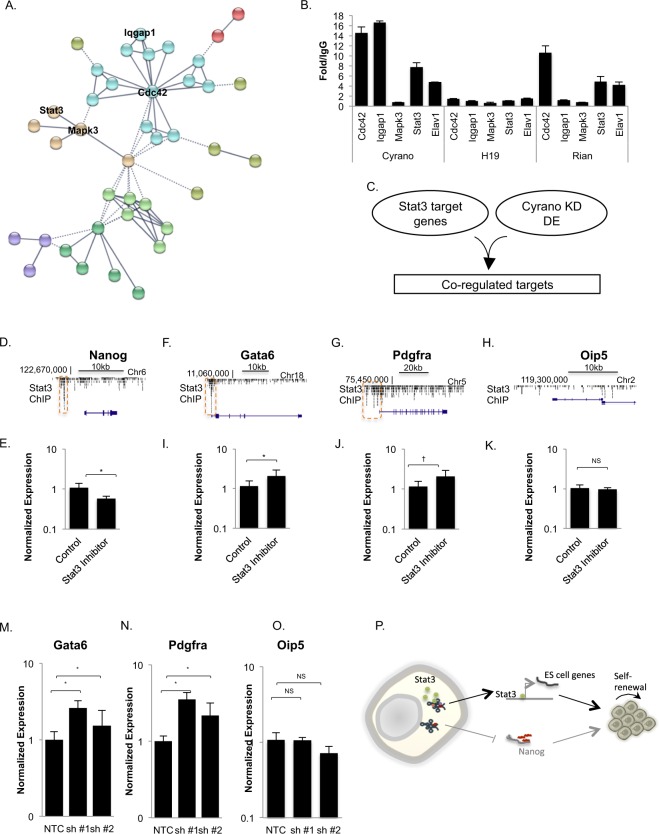
Cyrano interacts in a Developmental/Signaling Hub. (**A**) A sub-network containing developmentally important proteins identified by CHART was selected for further study. RNA immunoprecipitation for select proteins, along with the RNA binding protein, ELAV1 (Table S2) was used as an independent assay to investigate Cyrano binding (**B**), relative to lncRNAs H19 and Rian. Correlation analysis between Stat3 target genes and genes differentially expressed (DE) upon Cyrano depletion was carried out (**C**). (**D**) UCSC genome browser (mm8) view of Stat3 ChIP-Seq peaks^34^ (dashed box) upstream of Nanog. (**E**) The Stat3 inhibitor Stattic was used to investigate the effect of inhibition on Nanog levels. (**F,G**) UCSC genome browser view (mm8) of Stat3 ChIP-Seq peaks^34^ upstream of Gata6 and Pdgfra respectively, relative to the non-target, Oip5 (**H**). (**I**–**K**) The Stat3 inhibitor Stattic was used to examine the effect of Stat3 inhibition on Gata6 and Pdgfra levels respectively, relative to Oip5. (**M**–**O**) The levels of Stat3 target, early lineage specification genes Gata6 and Pdgfra were examined upon Cyrano depletion using independent shRNAs (Dharmacon), relative to the Cyrano neighboring gene, Oip5. ^*^p < 0.05, ^†^p < 0.1. (**P**) Prospective model of an additional Cyrano function elucidated based on the mass-spectrometry determined interactome.

CLIP data were from studies of numerous cell types, and the nature of the overlapping binding sites on Cyrano in the CLIP data (Fig. 2C) suggests that protein partners may be cell and/or context specific. Based on the previously observed loss-of-function phenotype in ES cells and evidence that Cyrano has developmentally relevant roles, we focused on the signaling/development network node for further study (Fig. 5A). Several members of this sub-network including STAT3, MAPK3 and CDC42 have been shown to be important developmentally. For instance, MAPK/ERK signaling is a central signaling axis that is important in the regulation of cell survival, which also has roles in differentiation processes from pluripotency towards lineage commitment^29,30^. STAT3 is the downstream transcription factor effector of the LIF/Stat3 signaling pathway, which is required for maintenance of self-renewal and pluripotency in mouse ES cells^31^. Emerging data support STAT3′s role as a lncRNA-binding protein, with lncRNA functioning to mediate STAT3′s subcellular transport and activation status^32,33^. We recently showed that Nanog levels decrease upon Cyrano depletion^17^. Nanog is downstream of the LIF/Stat3 signaling axis, which suggests that Cyrano interacting in a complex with Stat3 could be an additional mechanism by which Cyrano supports maintenance of Nanog levels.

We used the reverse independent assay, RNA immunoprecipitation to validate candidate interactors and found that select candidates CDC42, IQGAP1, STAT3 enriched for Cyrano (Fig. 5B), similar to the RNA binding protein ELAV1 (Table S2). However, unlike STAT3 (Fig. S2A), even though we were able to immunoprecipitate MAPK3/ERK1 (Fig. S2B), we were unable to validate MAPK3 interaction with Cyrano (Fig. 5B) in RNA immunoprecipitation experiments. Nevertheless, the successful validation of four of five interactors demonstrated the validity of our approach. Since STAT3 is central to ES cell maintenance, we sought to further determine whether the Cyrano-STAT3 interaction mediated downstream STAT3 function. We correlated STAT3 chromatin targets with genes that were differentially expressed with Cyrano depletion, and found that the pluripotency regulator NANOG is a STAT3 target that is downregulated with Cyrano depletion (Fig. 5C,D). Similar decreases in Nanog expression are observed with Cyrano shRNA-mediated knockdown^17^ and STAT3 inhibition (Fig. 5E). This suggests that Nanog regulation could be a mechanism where STAT3 and Cyrano converge to support ES cell maintenance. Similarly, select STAT3 targets (Gata6 and Pdgfra^34^; Fig. 5F,G) that direct lineage specification are responsive to STAT3 inhibition (Fig. 5I,J), relative to Oip5 (Fig. 5H–K), which was not found to be a STAT3 target gene^34^. Similarly, Gata6 and Pdgfra increase with Cyrano depletion (Figs 5M,N and S2C) when compared to Oip5 (Fig. 5O), a neighboring gene to Cyrano that is unaffected by its loss in ES cells^17^. Altogether, in addition to finding proteins with which Cyrano interact to mediate its function and/or regulation, we have used this unbiased approach to identify a prospective subset of interactions through which Cyrano supports ES cell maintenance (Fig. 5P).

## Discussion

Cyrano has previously been implicated as a central developmental regulator in zebrafish, and emerging roles in mammalian cells are indicative of functions in cell proliferation, cell adhesion and cell survival regulation in stem cells and cancer^17,18,35,36^. However, apart from a functional link to moderating *mir-7* function, mechanisms by which Cyrano functions to support ES cell maintenance remain enigmatic. Here, we expand the protein-interacting repertoire for Cyrano to provide a hypothesis-generating resource to examine the mechanisms used by Cyrano to regulate its targets, and those involved in regulating Cyrano itself.

The developmental/signaling sub-network, which led us to identify the Cyrano-STAT3 interaction, also contains proteins including IQGAP1 and CDC42, previously implicated in lncRNA signaling regulation. IQGAP1 is a scaffolding protein that interacts with various cellular proteins to modulate signaling pathways involved in cell adhesion, motility and cell proliferation, including in cooperation with the RhoGTPase CDC42^37^. Previous studies have demonstrated their interaction with the lncRNA *NRON*, where they function to regulate activity and trafficking of the transcription factor, NFAT^38,39^. This suggests that similar to NRON, one mechanism by which Cyrano could function may utilize an RNA-mediated scaffold or an RNA-protein transport mechanism. Such a scaffolding function could explain the potent effect Cyrano has on ES cell biology despite being present at only approximately 40 molecules per cell^17^. Such a large lncRNA molecule may adopt conformational structures that permit the docking of multiple proteins.

Candidates for nuclear functions include epigenetic regulators, including components of chromatin modifying and remodeling complexes, whose loss could be detrimental to the maintenance of self-renewing ES cell colonies^40^. Such interactions could explain Cyrano’s nuclear localization and extend Cyrano’s function to chromatin regulatory activities. Indeed, the human ortholog of Cyrano, has been found to contact chromatin^41^.

Overall, the Cyrano-protein interactome suggests that Cyrano functions alongside multiple protein components with established roles in regulating key processes for ES cell maintenance. ES cells are the *in vitro* representatives of the blastocyst-derived inner cell mass that self-renew and are able to differentiate into all cellular descendants of the three germ layers^11–13^. These central properties bestow significant potential for studying early developmental processes and for regenerative medicine for the treatment of various degenerative diseases. Key properties of pluripotent cells are directed by archetypal master signaling, transcription and epigenetic regulators, including NANOG, OCT4, STAT3 and MYC, which underpin the expression network of genes that support pluripotency and self-renewal and block those which promote lineage specification^42,43^. A supportive cast of regulators further bolsters the primary ES cell regulatory network by maintaining the characteristic cell cycle and apoptosis escape exhibited by ES cells^44–46^. Molecules such as Cyrano, which support cell survival and proliferation of ES cells, require further study to understand their individual contributions. It can be envisioned that these RNAs add a layer of specificity to established transcription factor-centered regulatory processes. In addition to the previously described *mir-7*-associated mechanism, the STAT3-based role described here is one potential method through which Cyrano could support self-renewal and pluripotency, to converge upon cooperative regulation of central regulators such as Nanog (Fig. 5P).

Various proteins identified in this study have not previously been identified as RBPs. For instance, while STAT3 has been found to interact with lncRNAs, it was not identified in proteomics screens for RBPs in ES cells. Importantly, it should be noted that methods such as CHART do not differentiate between direct and indirect interactors. Nevertheless, our validation demonstrates the utility of the method. For novel interactors, it will be important to define the directness and mode of interactions biochemically and functionally.

Affinity-proteomics approaches may also miss previously identified interactions, as well as deduced interactions from known functions. For example, only one component of the RISC complexes was identified in our studies, yet Cyrano is known to interact with miRNAs^17,23^. Finally, the function of a particular interaction may require context specific candidate validation in a particular process, such as cell cycle regulation or escape from apoptosis. Nonetheless, these candidates provide a resource for further mechanistic evaluation of Cyrano function.

## Materials and Methods

### Cell Culture

Mouse R1 ES cells (XY^47^, were maintained in Dulbecco’s Modified Eagle Medium supplemented with 10% FBS, 10% Knockout Serum Replacement, 2mM L-glutamate, 1 mM sodium pyruvate, 0.1 mM β-mercaptoethanol, and 100U/ml penicillin-streptomycin and LIF on gelatin-coated dishes. Stattic (2 μM) was added to cells for 4–6 hours to inhibit STAT3 activity where indicated. shRNA constructs targeting Cyrano and non-targeting control (Dharmacon) were transfected into ESCs using Lipofectamine LTX reagent (ThermoFisher) according to the manufacturer’s instructions, followed by puromycin selection to enrich for transfectants.

### Capture Hybridization Analysis of RNA Targets (CHART)

CHART enrichment was performed generally as previously described^25,26^. CHART extract was prepared from approximately 2.5 × 10^7^ cells, fixed with formaldehyde (1%, 10 min; 3%, 30 min). Lysate was divided into 75ul reactions and hybridized with optimized amounts^25,26^ of biotinylated complementary, sense or scrambled oligonucleotide cocktail (Supplementary Table S3) overnight at room temperature. Complexes were captured using 80 μl MyOneC1 streptavidin beads (Invitrogen) overnight at room temperature with rotation. Bound material was extensively washed and eluted using RNase H (New England Biolabs) for 30 min at room temperature. Crosslinks were reversed as described in^25,26^ and RNA purified from 1/5 total sample volume using the Qiagen RNeasy kit while proteins from the remaining pooled sample were precipitated with trichloroacetic acid, resuspended and run on an SDS-PAGE system to separate proteins from lower molecular weight contaminants, and the entire protein region of the gel excised at processed at the Proteomics and Mass Spectrometry Facility, University of Massachusetts Medical School. Gel fragments were subjected to in-gel trypsin digestion after reduction with DTT and alkylation with IAA. Peptides eluted from the gel were lyophilized and re-suspended in 25 µL of 5% acetonitrile (0.1% (v/v) TFA). A 3 µL injection was loaded by a Waters NanoAcquity UPLC in 5% acetonitrile (0.1% formic acid) at 4.0 µL/min for 4.0 min onto a 100 µm I.D. fused-silica pre-column packed with 2 cm of 5 µm (200 Å) Magic C18AQ (Bruker-Michrom). Peptides were eluted at 300 nL/min from a 75 µm I.D. gravity-pulled analytical column packed with 25 cm of 3 µm (100 Å) Magic C18AQ particles using a linear gradient from 5–35% of mobile phase B (acetonitrile + 0.1% formic acid) in mobile phase A (water + 0.1% formic acid) over 90 minutes. Ions were introduced by positive electrospray ionization via liquid junction at 1.3 kV into a Thermo Scientific Q Exactive hybrid mass spectrometer. Mass spectra were acquired over *m/z* 300–1750 at 70,000 resolution (*m/z* 200) with an AGC target of 1e6, and data-dependent acquisition selected the top 10 most abundant precursor ions for tandem mass spectrometry by HCD fragmentation using an isolation width of 1.6 Da, max fill time of 110 ms, and AGC target of 1e5. Peptides were fragmented by a normalized collisional energy of 27, and fragment spectra acquired at a resolution of 17,500 (*m/z* 200). Raw data files were peak processed with Proteome Discoverer (version 1.4, Thermo) followed by identification using Mascot Server (version 2.5, Matrix Science) against the *Mus musculus* Uniprot FASTA file (downloaded 1/2015). Search parameters included Trypsin/P specificity, up to 2 missed cleavages, a fixed modification of carbamidomethyl cysteine, and variable modifications of oxidized methionine, pyroglutamic acid for Q, and N-terminal acetylation. Assignments were made using a 10 ppm mass tolerance for the precursor and 0.05 Da mass tolerance for the fragments. All non-filtered search results were processed by Scaffold (version 4.4.4, Proteome Software, Inc.) utilizing the Trans-Proteomic Pipeline (Institute for Systems Biology) with threshold values set at 80% probability for peptides (1.0% false-discovery rate) and 90% for proteins, and quantitative comparisons made using the iBAQ quantitation method with all samples normalized by total ion current for the run. Single peptide hits^48–50^ that were unique to the Cyrano pulldown only, were also included for network generation.

### RNA Extraction and Quantitative RT-PCR

Total RNA was isolated using the Quick-RNA MiniPrep Kit (Zymo), reverse transcription was carried out using the iScript reagent (Bio-Rad) and qRT-PCR for lncRNA and mRNA transcripts was performed using primers listed (Supplementary Table S4).

### RNA Immunoprecipitation (RIP)

RIP was carried out as described^51^. Briefly, 10–20 million cells were fixed with 0.3% formaldehyde for 30 minutes on ice, followed by the addition of glycine for 5 minutes at room temperature. RIPA (50 mM Tris (pH 8), 150 mM KCl, 0.1% SDS, 1% Triton-X, 5 mM EDTA, 0.5% sodium deoxycholate, and freshly added 0.5 mM DTT, protease inhibitor cocktail and RNasin), and brief sonication (Bioruptor: 2 × 30 second; 1 minute off cycles) was used for cell lysis. The lysate was centrifuged at 4 °C max speed for 10 minutes, and the supernatant collected and diluted with an equal volume of fRIP binding/wash buffer (150 mM KCl, 25 mM Tris, 5 mM EDTA, 0.5% NP-40 and freshly added 0.5 mM DTT, protease inhibitors, and RNasin). Diluted lysate was precleared with 50 μl beads for 30 minutes followed by the addition of 8 μg pre-coupled Dynabeads-Stat3 antibody (C-20, Santa Cruz), Iqgap1 (D6E3J, Cell Signaling Technologies), Cdc42 (EPR15620, Abcam), Elav1 (D9W7E, Cell Signaling Technologies), Erk1/Mapk3 (137F5, Cell Signaling Technologies) or IgG control. Antibody coupled beads and lysate were rotated overnight at 4 °C followed by washing four times with 1 mL of fRIP binding/washing buffer with protease and RNase inhibitors. Crosslinks were reversed in the presence of proteinase K, and RNA purified using TriZol followed by the Zymo RNA Clean and Purification Kit. Eluted RNA was subjected to qPCR to detect RNAs bound by Stat3.

### Western blot Analysis

Cell extracts were prepared using a modified RIPA buffer (50 mM Tris pH 8.0, 150 mM NaCl, 1% NP40, 0.5% deoxycholate, 0.1% SDS, dithothreitol) containing protease inhibitors (Roche), followed by electrophoresis and blotting on to a nitrocellulose/PVDF membrane (BioRad) before incubation in the appropriate primary antibody. After incubation with HRP-conjugated secondary antibody (Santa Cruz), membranes were developed with SuperSignal West Dura Chemiluminescent substrate (ThermoFisher).

### Bioinformatics

Transcript length, exon count and exon size statistics were computed using the Gencode GTF annotations for mouse, vM15, and human, v27^19,52,53^. We parsed the GTF files using custom Python scripts based on the HTSeq package^54^ and graphs were drawn using R^55^. eCLIP and CLIPdb analyses were performed using data from^22^ using BigBed tools^56^ and custom Python scripts. Scripts and data used for analyses can be found here: https://github.com/starmerj/cyrano_interactome.

## Electronic supplementary material

Supplementary Figures and Tables

Supplementary Table 3
