# Supplementary material for: Interactome determination of a Long Noncoding RNA implicated in Embryonic Stem Cell Self-Renewal
Source: Sci Rep. 2018 Dec 4;8:17568. doi: 10.1038/s41598-018-34864-z (PMC6279841; doi:10.1038/s41598-018-34864-z)

Supplementary Figure Legends

Supplemental Figure 1. Cyrano-Protein Network in ES cells. STRING-generated networks analysis showing protein names.

Supplemental Figure 2. Assessment of the Cyrano-network. Immunoprecipitation for Stat3 followed by Western blotting to compare isolation of Stat3 by the Stat3 antibody relative to IgG (A), and isolation of Erk by the Erk antibody relative to IgG (B). qRT-PCR analysis after Cyrano knockdown using two independent shRNAs, relative to a non-targeting control.

Reference for Supplementary Tables 1-2

Pardo, M. & Choudhary, J. S. Assignment of Protein Interactions from Affinity Purification/Mass Spectrometry Data. *J. Proteome Res.* **11,** 1462–1474 (2012)

Supplementary Table S1. Proteins with 2-peptide hits from Cyrano CHART-Mass Spectrometry

			**Fold Change (Cyrano CHART)**		**Protein identification probability**		
Symbol	**Accession**	**Name**	**Rel. to Sen oligo**	**Rel. to Scr oligo**	**Sen oligo**	**Scr oligo**	**Cyr-ano**
Abce1	P61222	ATP-binding cassette sub-family E member 1	INF	INF	0	0	100%
Actr1a	P61164	Alpha-centractin	INF	87	0	93%	100%
Actr2	P61161	Actin-related protein 2	INF	74	0	100%	100%
Ahnak2	F7CVJ5	Protein Ahnak2 (Fragment)	INF	INF	0	0	100%
Ak2	Q9WTP6-2	Isoform 2 of Adenylate kinase 2, mitochondrial	INF	12	0	99%	100%
Akr1b7	P21300	Aldose reductase-related protein 1	9.4	15	27%	26%	100%
Alyref	O08583	THO complex subunit 4	INF	INF	0	0	100%
Arcn1	Q5XJY5	Coatomer subunit delta	INF	26	0	99%	100%
Arhgdia	Q99PT1	Rho GDP-dissociation inhibitor 1	INF	INF	0	0	100%
Arpc5	Q9CPW4	Actin-related protein 2/3 complex subunit 5	INF	INF	0	0	100%
Arpc5l	Q9D898	Actin-related protein 2/3 complex subunit 5-like protein	5.9	INF	99%	99%	100%
Atp2a1	Q8R429	Sarcoplasmic/endoplasmic reticulum calcium ATPase 1	INF	INF	0	6%	98%
Atp6v1g1	Q9CR51	V-type proton ATPase subunit G 1	INF	6.6	0	100%	100%
Btf3	Q64152-2	Isoform 2 of Transcription factor BTF3	INF	INF	0	0	100%
Calml3	Q9D6P8	Calmodulin-like protein 3	INF	INF	6%	68%	98%
Cap1	P40124	Adenylyl cyclase-associated protein 1	INF	72	0	99%	100%
Capns1	O88456	Calpain small subunit 1	INF	INF	0	85%	98%
Cbr1	P48758	Carbonyl reductase [NADPH] 1	INF	7.4	0	100%	100%
Cct6a	P80317	T-complex protein 1 subunit zeta	7.8	42	100%	97%	100%
Cct7	P80313	T-complex protein 1 subunit eta	5.5	18	99%	99%	100%
Cdc42	P60766	Cell division control protein 42 homolog	INF	200	0	99%	100%
Clic1	Q9Z1Q5	Chloride intracellular channel protein 1	INF	10	0	100%	100%
Cltb	Q6IRU5-2	Isoform 2 of Clathrin light chain B	INF	INF	0	0	100%
Cndp2	Q9D1A2	Cytosolic non-specific dipeptidase	INF	INF	0	0	100%
Copb1	Q9JIF7	Coatomer subunit beta	INF	8.3	0	99%	100%
Csde1	Q91W50	Cold shock domain-containing protein E1	INF	INF	0	0	100%
Cse1l	Q9ERK4	Exportin-2	INF	INF	0	0	100%
Ctnna1	P26231	Catenin alpha-1	INF	INF	0	0	100%
Dctn2	Q99KJ8	Dynactin subunit 2	INF	14	0	99%	100%
Dnajb1	Q9QYJ3	DnaJ homolog subfamily B member 1	INF	INF	0	0	100%
Dnm1l	Q8K1M6-2	Isoform 2 of Dynamin-1-like protein	INF	INF	0	0	100%
Dync1i2	O88487	Cytoplasmic dynein 1 intermediate chain 2	INF	56	0	99%	100%
Eef1b	O70251	Elongation factor 1-beta	5.5	6.5	99%	100%	100%
Eef1d	P57776	Elongation factor 1-delta	24	INF	83%	90%	100%
Eif1	P48024	Eukaryotic translation initiation factor 1	INF	INF	0	0	100%
Eif3a	P23116	Eukaryotic translation initiation factor 3 subunit A	INF	INF	0	0	100%
Eif3c	Q8R1B4	Eukaryotic translation initiation factor 3 subunit C	INF	INF	0	0	100%
Eif3e	P60229	Eukaryotic translation initiation factor 3 subunit E	5.4	40	100%	100%	100%
Eif3h	Q91WK2	Eukaryotic translation initiation factor 3 subunit H	INF	7.9	0	99%	100%
Eif3i	Q9QZD9	Eukaryotic translation initiation factor 3 subunit I	INF	INF	0	0	100%
Eif3k	Q9DBZ5	Eukaryotic translation initiation factor 3 subunit K	INF	INF	0	0	100%
Eif4b	Q8BGD9	Eukaryotic translation initiation factor 4B	INF	INF	0	0	100%
Eif4g1	Q6NZJ6	Eukaryotic translation initiation factor 4 gamma 1	INF	INF	0	0	100%
Eno1	P17182	Alpha-enolase	26	61	100%	100%	100%
Etf1	Q8BWY3	Eukaryotic peptide chain release factor subunit 1	INF	INF	0	0	100%
Farsb	Q9WUA2	Phenylalanine--tRNA ligase beta subunit	INF	INF	0	0	100%
Fbl	P35550	rRNA 2'-O-methyltransferase fibrillarin	INF	INF	0	0	100%
Fgb	Q8K0E8	Fibrinogen beta chain	INF	INF	0	0	100%
Fkbp3	Q62446	Peptidyl-prolyl cis-trans isomerase FKBP3	INF	INF	0	0	100%
Flnb	Q80X90	Filamin-B	INF	5.3	0	100%	100%
Fubp1	Q91WJ8-2	Isoform 2 of Far upstream element-binding protein 1	INF	INF	0	0	100%
Gdi1	P50396	Rab GDP dissociation inhibitor alpha	INF	15	0	100%	100%

Gdi2	Q61598-2	Isoform 2 of Rab GDP dissociation inhibitor beta	INF	30	0	97%	100%
Gm15013	V9GWY0	40S ribosomal protein S4	INF	INF	0	0	100%
H1f0	P10922	Histone H1.0	INF	INF	0	0	100%
H2afy	Q9QZQ8-2	Isoform 1 of Core histone macro-H2A.1	INF	INF	0	0	100%
Hba	P01942	Hemoglobin subunit alpha	5.6	100	100%	99%	100%
Hbb-b1	P02088	Hemoglobin subunit beta-1	INF	INF	0	0	100%
Hbb-y	P02104	Hemoglobin subunit epsilon-Y2	INF	INF	0	0	98%
Hdgf	P51859	Hepatoma-derived growth factor	INF	INF	0	0	100%
Hdlbp	Q8VDJ3	Vigilin	INF	15	0	95%	100%
Hist1h1b	P43276	Histone H1.5	INF	420	0	75%	100%
Hist1h1d	P43277	Histone H1.3	20	110	73%	93%	100%
Hist1h1e	P43274	Histone H1.4	17	74	79%	100%	100%
Hk1	P17710-2	Isoform HK1-SB of Hexokinase-1	INF	7.4	0	95%	100%
Hmga1	P17095	High mobility group protein HMG-I/HMG-Y	INF	INF	0	0	100%
Hmgb1	P63158	High mobility group protein B1	INF	610	0	97%	100%
Hnrnpab	Q99020	Heterogeneous nuclear ribonucleoprotein A/B	INF	INF	0	0	100%
Hnrnpc	Q9Z204-2	Isoform C1 of Heterogeneous nuclear ribonucleoproteins C1/C2	INF	19	0	100%	100%
Hnrnpdl	Q9Z130	Heterogeneous nuclear ribonucleoprotein D-like	INF	INF	0	0	98%
Hnrnpm	Q9D0E1-2	Isoform 2 of Heterogeneous nuclear ribonucleoprotein M	9.6	13	100%	100%	100%
Hnrnpr	F7B5B5	Protein Hnrnpr	INF	21	0	99%	98%
Hnrnpu	Q8VEK3	Heterogeneous nuclear ribonucleoprotein U	INF	25	0	100%	100%
Hspa1l	P16627	Heat shock 70 kDa protein 1-like	INF	5.4	0	100%	100%
Hspa4	Q61316	Heat shock 70 kDa protein 4	INF	INF	0	0	100%
Hspa4l	P48722-2	Isoform 2 of Heat shock 70 kDa protein 4L	INF	INF	0	0	100%
Hspa9	P38647	Stress-70 protein, mitochondrial	40	24	99%	100%	100%
Hyou1	Q9JKR6	Hypoxia up-regulated protein 1	INF	INF	0	0	100%
Idh1	O88844	Isocitrate dehydrogenase [NADP] cytoplasmic	INF	INF	0	0	100%
Ilf2	Q9CXY6	Interleukin enhancer-binding factor 2	INF	INF	0	0	100%
Ilf3	Q9Z1X4-2	Isoform 2 of Interleukin enhancer-binding factor 3	INF	INF	0	0	100%
Ipo7	Q9EPL8	Importin-7	INF	INF	0	0	100%
Iqgap1	Q9JKF1	Ras GTPase-activating-like protein IQGAP1	INF	6.5	96%	100%	100%
Itih2	Q61703	Inter-alpha-trypsin inhibitor heavy chain H2	INF	INF	0	0	100%
Khsrp	Q3U0V1	Far upstream element-binding protein 2	INF	INF	0	0	100%
Kif5b	Q61768	Kinesin-1 heavy chain	INF	35	0	69%	100%
Letm1	Q9Z2I0	LETM1 and EF-hand domain-containing protein 1, mitochondrial	INF	INF	0	0	100%
Lmnb1	P14733	Lamin-B1	INF	INF	0	0	100%
Mapk3	Q63844	Mitogen-activated protein kinase 3	INF	INF	0	0	100%
Matr3	Q8K310	Matrin-3	INF	INF	0	0	100%
Mcts1	Q9DB27-2	Isoform 2 of Malignant T-cell-amplified sequence 1	INF	INF	0	0	100%
Morc2a	Q69ZX6	MORC family CW-type zinc finger protein 2A	INF	INF	0	0	100%
Myl12b	Q3THE2	Myosin regulatory light chain 12B	16	34	64%	99%	100%
Nampt	Q99KQ4	Nicotinamide phosphoribosyltransferase	21	5.1	99%	100%	100%
Nap1l1	P28656	Nucleosome assembly protein 1-like 1	INF	INF	0	0	100%
Ndufa4	Q62425	Cytochrome c oxidase subunit NDUFA4	INF	INF	0	0	100%
Nutf2	P61971	Nuclear transport factor 2	INF	6.6	0	100%	100%
Pa2g4	P50580	Proliferation-associated protein 2G4	36	23	99%	100%	100%
Pdcd10	Q8VE70	Programmed cell death protein 10	INF	INF	0	0	100%
Pdia3	P27773	Protein disulfide-isomerase A3	INF	21	0	100%	100%
Pdia6	Q922R8	Protein disulfide-isomerase A6	9.2	INF	99%	0	100%
Pdlim1	O70400	PDZ and LIM domain protein 1	INF	INF	0	0	100%
Pls3	Q99K51	Plastin-3	INF	5.9	99%	100%	100%
Ppa1	Q9D819	Inorganic pyrophosphatase	INF	INF	0	0	100%
Ppia	P17742	Peptidyl-prolyl cis-trans isomerase A	16	14	100%	100%	100%
Ppp2cb	P62715	Serine/threonine-protein phosphatase 2A catalytic subunit beta isoform	INF	11	0	99%	100%
Prdx4	O08807	Peroxiredoxin-4	INF	INF	0	0	99%
Psmc2	P46471	26S protease regulatory subunit 7	INF	INF	0	98%	100%
Psmc6	P62334	26S protease regulatory subunit 10B	INF	INF	0	0	100%
Psmd11	Q8BG32	26S proteasome non-ATPase regulatory subunit 11	INF	11	0	99%	100%
Psmd6	Q99JI4	26S proteasome non-ATPase regulatory subunit 6	INF	8.3	0	100%	98%

Psme2	P97372	Proteasome activator complex subunit 2	INF	INF	0	0	100%
Psme3	A2A4J3	Proteasome activator complex subunit 3 (Fragment)	INF	INF	0	0	100%
Ptbp1	P17225	Polypyrimidine tract-binding protein 1	INF	8.3	0	98%	100%
Ptma	P26350	Prothymosin alpha	20	INF	99%	0	100%
Rab11b	P46638	Ras-related protein Rab-11B	7.2	11	100%	100%	100%
Rab18	P35293	Ras-related protein Rab-18	INF	INF	0	0	100%
Rab25	Q9WTL2	Ras-related protein Rab-25	INF	INF	0	0	100%
Rab2a	P53994	Ras-related protein Rab-2A	INF	7.7	0	100%	100%
Rab6a	P35279-2	Isoform 2 of Ras-related protein Rab-6A	INF	INF	0	0	100%
Rab7a	P51150	Ras-related protein Rab-7a	12	7.4	89%	100%	100%
Ran	P62827	GTP-binding nuclear protein Ran	19	150	100%	100%	100%
Rangap1	P46061	Ran GTPase-activating protein 1	INF	INF	0	0	100%
Rap1a	P62835	Ras-related protein Rap-1A	INF	5.8	0	99%	100%
Rbbp4	Q60972	Histone-binding protein RBBP4	INF	INF	0	0	100%
Rbmx	Q9WV02-2	Isoform 2 of RNA-binding motif protein, X chromosome	INF	INF	0	0	100%
Rbx1	P62878	E3 ubiquitin-protein ligase RBX1	INF	INF	0	0	100%
Rpl10	Q6ZWV3	60S ribosomal protein L10	INF	INF	0	0	100%
Rpl10a	P53026	60S ribosomal protein L10a	INF	27	0	100%	100%
Rpl12	P35979	60S ribosomal protein L12	9	14	100%	100%	100%
Rpl13	P47963	60S ribosomal protein L13	7.9	INF	98%	16%	100%
Rpl13a	P19253	60S ribosomal protein L13a	INF	10	0	99%	100%
Rpl17	Q9CPR4	60S ribosomal protein L17	INF	38	0	96%	100%
Rpl19	P84099	60S ribosomal protein L19	INF	7.1	0	99%	100%
Rpl21	O09167	60S ribosomal protein L21	INF	INF	0	0	100%
Rpl26	P61255	60S ribosomal protein L26	INF	INF	0	0	100%
Rpl3	P27659	60S ribosomal protein L3	INF	180	0	99%	100%
Rpl32	P62911	60S ribosomal protein L32	INF	INF	0	0	100%
Rpl34	Q9D1R9	60S ribosomal protein L34	INF	INF	0	0	100%
Rpl35	Q6ZWV7	60S ribosomal protein L35	INF	36	0	99%	100%
Rpl36	Q6ZWZ4	60S ribosomal protein L36	7.4	24	100%	99%	100%
Rpl38	Q9JJI8	60S ribosomal protein L38	6.3	INF	98%	0	100%
Rpl4	Q9D8E6	60S ribosomal protein L4	INF	27	0	100%	100%
Rpl5	P47962	60S ribosomal protein L5	INF	INF	0	0	100%
Rpl6	P47911	60S ribosomal protein L6	14	18	100%	100%	100%
Rpl7	F6XI62	60S ribosomal protein L7 (Fragment)	INF	75	0	98%	100%
Rpl7a	P12970	60S ribosomal protein L7a	7.9	88	100%	100%	100%
Rpl8	P62918	60S ribosomal protein L8	INF	140	0	99%	100%
Rpl9	P51410	60S ribosomal protein L9	INF	9.8	0	99%	100%
Rps11	P62281	40S ribosomal protein S11	INF	13	0	100%	100%
Rps12	P63323	40S ribosomal protein S12	INF	66	0	100%	100%
Rps13	P62301	40S ribosomal protein S13	23	14	99%	100%	100%
Rps14	P62264	40S ribosomal protein S14	26	INF	98%	0	100%
Rps15	P62843	40S ribosomal protein S15	INF	INF	0	0	100%
Rps17	P63276	40S ribosomal protein S17	INF	25	0	99%	100%
Rps18	P62270	40S ribosomal protein S18	8.6	15	95%	100%	100%
Rps19	Q9CZX8	40S ribosomal protein S19	18	270	100%	99%	100%
Rps20	P60867	40S ribosomal protein S20	INF	9	0	100%	100%
Rps21	Q9CQR2	40S ribosomal protein S21	5	INF	99%	0	100%
Rps23	P62267	40S ribosomal protein S23	INF	INF	0	0	100%
Rps24	P62849-2	Isoform 2 of 40S ribosomal protein S24	INF	7.1	0	100%	100%
Rps3a	P97351	40S ribosomal protein S3a	INF	22	0	99%	100%
Rps4x	P62702	40S ribosomal protein S4, X isoform	11	21	99%	100%	100%
Rps5	P97461	40S ribosomal protein S5	13	24	100%	100%	100%
Rps6	P62754	40S ribosomal protein S6	8.2	20	100%	100%	100%
Rps7	P62082	40S ribosomal protein S7	INF	24	0	99%	100%
Rtcb	Q99LF4	tRNA-splicing ligase RtcB homolog	INF	INF	0	0	100%
Ruvbl1	P60122	RuvB-like 1	INF	INF	0	0	100%
Sars	P26638	Serine--tRNA ligase, cytoplasmic	INF	INF	0	0	100%
Sept9	Q80UG5-3	Isoform 3 of Septin-9	INF	28	0	64%	100%
Serbp1	Q9CY58-2	Isoform 2 of Plasminogen activator inhibitor 1 RNA-binding protein	INF	INF	0	0	100%

Sf1	Q64213-2	Isoform CW17E of Splicing factor 1	INF	INF	0	0	100%
Sf3a3	Q9D554	Splicing factor 3A subunit 3	INF	INF	0	0	100%
Sfn	O70456	14-3-3 protein sigma	5.6	6	100%	100%	100%
Sfpq	Q8VIJ6	Splicing factor, proline- and glutamine-rich	11	12	64%	100%	100%
Sh3bgrl3	Q91VW3	SH3 domain-binding glutamic acid-rich-like protein 3	INF	INF	0	0	100%
Sh3gl1	Q62419	Endophilin-A2	INF	110	0	68%	100%
Snd1	Q78PY7	Staphylococcal nuclease domain-containing protein 1	INF	6.1	0	100%	100%
Snx6	Q6P8X1	Sorting nexin-6	INF	INF	0	0	100%
Spag9	Q58A65-2	Isoform 2 of C-Jun-amino-terminal kinase-interacting protein 4	INF	INF	0	0	100%
Sptbn1	Q62261	Spectrin beta chain, non-erythrocytic 1	INF	INF	0	0	100%
Sptbn2	Q68FG2	Protein Sptbn2	INF	INF	0	8%	100%
Srp68	Q8BMA6	Signal recognition particle subunit SRP68	INF	INF	0	0	100%
Srsf1	Q6PDM2-2	Isoform 2 of Serine/arginine-rich splicing factor 1	INF	INF	0	0	100%
Srsf2	Q62093	Serine/arginine-rich splicing factor 2	5.7	INF	100%	99%	100%
Srsf3	P84104-2	Isoform Short of Serine/arginine-rich splicing factor 3	INF	INF	0	0	100%
Srsf7	Q8BL97-2	Isoform 2 of Serine/arginine-rich splicing factor 7	INF	INF	0	0	100%
Stat3	P42227-3	Isoform Del-701 of Signal transducer and activator of transcription 3	INF	6.9	0	99%	100%
Strap	Q9Z1Z2	Serine-threonine kinase receptor-associated protein	INF	INF	0	0	100%
Syncrip	Q7TMK9-2	Isoform 2 of Heterogeneous nuclear ribonucleoprotein Q	INF	INF	0	0	100%
Thrap3	Q569Z6	Thyroid hormone receptor-associated protein 3	INF	INF	0	0	100%
Timm8b	P62077	Mitochondrial import inner membrane translocase subunit Tim8 B	INF	INF	0	0	100%
Tkt	P40142	Transketolase	INF	5.3	94%	98%	100%
Tln2	Q71LX4	Talin-2	INF	INF	0	0	98%
Tmod3	Q9JHJ0	Tropomodulin-3	INF	INF	0	0	100%
Tmsb4x	P20065-2	Isoform Short of Thymosin beta-4	INF	INF	0	0	100%
Tpd52	Q62393-2	Isoform 2 of Tumor protein D52	INF	INF	0	0	100%
Tppp3	Q9CRB6	Tubulin polymerization-promoting protein family member 3	INF	INF	0	0	100%
Trim29	Q8R2Q0	Tripartite motif-containing protein 29	INF	14	0	100%	100%
Tuba4a	A0A087WSL5	Tubulin alpha-4A chain (Fragment)	INF	5	0	8%	100%
Uba1	Q02053	Ubiquitin-like modifier-activating enzyme 1	INF	8.1	0	100%	100%
Ube2v2	Q9D2M8-2	Isoform 2 of Ubiquitin-conjugating enzyme E2 variant 2	INF	INF	0	0	100%
Usp36	B1AQJ2	Ubiquitin carboxyl-terminal hydrolase 36	INF	INF	0	0	99%
Usp5	P56399	Ubiquitin carboxyl-terminal hydrolase 5	INF	7.2	0	100%	100%
Vim	P20152	Vimentin	6.5	41	100%	100%	100%
Wdr1	O88342	WD repeat-containing protein 1	INF	22	0	100%	100%
Ybx1	P62960	Nuclease-sensitive element-binding protein 1	INF	INF	0	0	100%
Ybx3	Q9JKB3-2	Isoform 2 of Y-box-binding protein 3	INF	INF	0	0	100%
Ywhah	P68510	14-3-3 protein eta	INF	INF	0	0	100%

† Grey text: Common contaminant families of proteins [57] not used for clarity in STRING network generation.

Supplementary Table S2. Proteins with unique single-peptide hits from Cyrano CHART-Mass Spectrometry

Gene Name	Accession	Protein name
Abca17	E9PX95	ATP-binding cassette sub-family A member 17
Acat2	Q8CAY6	Acetyl-CoA acetyltransferase, cytosolic
Actl6a	Q9Z2N8	Actin-like protein 6A (53 kDa BRG1-associated factor A)
Adrm1	Q9JKV1	Proteasomal ubiquitin receptor
Adss	P46664	Adenylosuccinate synthetase isozyme 2
Crybg1	E9PVP1	Crystallin beta-gamma domain-containing 1
Akt1s1	Q9D1F4	Proline-rich AKT1 substrate 1
Anp32e	P97822	Acidic leucine-rich nuclear phosphoprotein 32 family member E
Ap1b1	O35643	AP-1 complex subunit beta-1
Apex1	P28352	DNA-(apurinic or apyrimidinic site) lyase
Api5	O35841	Apoptosis inhibitor 5
Apob	E9Q414	Apolipoprotein B-100
Arpc1b	Q9WV32	Actin-related protein 2/3 complex subunit 1B
Atic	Q9CWJ9	Bifunctional purine biosynthesis protein PURH
Atp5c1	Q91VR2	ATP synthase subunit gamma, mitochondrial
Atp5o	Q9DB20	ATP synthase subunit O, mitochondrial
Bicdl2	Q8CHW5	BICD family-like cargo adapter 2
Btf3l4	Q9CQH7	Transcription factor BTF3 homolog 4
C4b	P01029	Complement C4-B
Ca2	P00920	Carbonic anhydrase 2
Cand1	Q6ZQ38	Cullin-associated NEDD8-dissociated protein 1
Capg	P24452	Macrophage-capping protein
Capza1	P47753	F-actin-capping protein subunit alpha-1
Cbx3	P23198	Chromobox protein homolog 3
Ccdc22	Q9JIG7	Coiled-coil domain-containing protein 22
Ccdc6	D3YZP9	Coiled-coil domain-containing protein 6
Cdh1	P09803	Cadherin-1 (ARC-1) (Epithelial cadherin)
Chmp1a	Q921W0	Charged multivesicular body protein 1a
Chmp2a	Q9DB34	Charged multivesicular body protein 2a
Cirbp	P60824	Cold-inducible RNA-binding protein
Clpp	O88696	ATP-dependent Clp protease proteolytic subunit
Cluh	Q5SW19	Clustered mitochondria protein homolog
Cnn2	Q08093	Calponin-2
Copa	Q8CIE6	Coatomer subunit alpha
Cops3	O88543	COP9 signalosome complex subunit 3
Copz1	P61924	Coatomer subunit zeta-1
Cpsf7	Q8BTV2	Cleavage and polyadenylation specificity factor subunit 7
Csrp1	P97315	Cysteine and glycine-rich protein 1
Cyb5r3	Q9DCN2	NADH-cytochrome b5 reductase 3
Cyfip1	Q7TMB8	Cytoplasmic FMR1-interacting protein 1
Ddx21	Q9JIK5	Nucleolar RNA helicase 2
Ddx6	P54823	Probable ATP-dependent RNA helicase
Dhx15	O35286	Pre-mRNA-splicing factor ATP-dependent RNA helicase
Dlg1	Q811D0	Disks large homolog 1

Dnaja2	Q9QYJ0	DnaJ homolog subfamily A member 2
Dpysl2	O08553	Dihydropyrimidinase-related protein 2
Dr1	Q91WV0	Down-regulator of transcription 1
Dsg3	O35902	Desmoglein-3
Dynll1	P63168	Dynein light chain 1, cytoplasmic
Dynlrb2	Q9DAJ5	Dynein light chain roadblock-type 2
Edf1	Q9JMG1	Endothelial differentiation-related factor 1
Efcab3	Q80X60	EF-hand calcium-binding domain-containing protein 3
Eftud2	O08810	116 kDa U5 small nuclear ribonucleoprotein component
Ehd1	Q9WVK4	EH domain-containing protein 1
Eif1a	Q60872	Eukaryotic translation initiation factor 1A
Eif2s2	Q99L45	Eukaryotic translation initiation factor 2 subunit 2
Eif3j1	Q3UGC7	Eukaryotic translation initiation factor 3 subunit J-A
Eif3m	Q99JX4	Eukaryotic translation initiation factor 3 subunit M
Eif4e	P63073	Eukaryotic translation initiation factor 4E
Elavl1	P70372	ELAV-like protein 1
Esd	Q9R0P3	S-formylglutathione hydrolase
Esrp1	Q3US41	Epithelial splicing regulatory protein 1
Etfa	Q99LC5	Electron transfer flavoprotein subunit alpha
Farsa	Q8C0C7	Phenylalanine--tRNA ligase alpha subunit
Fau	P62862	40S ribosomal protein S30
Fgg	Q8VCM7	Fibrinogen gamma chain
Fkbp1a	P26883	Peptidyl-prolyl cis-trans isomerase
Furin	P23188	Furin
Fus	G3UXT7	RNA-binding protein FUS
G3bp1	P97855	Ras GTPase-activating protein-binding protein 1
G6pdx	Q00612	Glucose-6-phosphate 1-dehydrogenase X
Gigyf2	Q6Y7W8	GRB10-interacting GYF protein 2
Gmps	Q3THK7	GMP synthase
Gnb1	H3BKR2	Guanine nucleotide-binding protein
Gspt1	Q8R050	Eukaryotic peptide chain release factor GTP-binding subunit
Gstp1	P19157	Glutathione S-transferase P 1
Gtf2f2	Q8R0A0	General transcription factor IIF subunit 2
Hadh	Q61425	Hydroxyacyl-coenzyme A dehydrogenase, mitochondrial
Hectd1	Q69ZR2	E3 ubiquitin-protein ligase
Hmox2	O70252	Heme oxygenase 2
Hnrnpl	Q8R081	Heterogeneous nuclear ribonucleoprotein L
Hypk	Q9CR41	Huntingtin-interacting protein K
Igfbp3	P47878	Insulin-like growth factor-binding protein 3
Kars	Q99MN1	Lysine--tRNA ligase
Khdrbs1	Q60749	KH domain-containing, RNA-binding, signal transduction-associated protein 1
Lamtor1	Q9CQ22	Ragulator complex protein LAMTOR1
Lars	Q8BMJ2	Leucine--tRNA ligase, cytoplasmic
Ldhb	P16125	L-lactate dehydrogenase B chain
Lemd2	Q6DVA0	LEM domain-containing protein 2
Lrrc59	Q922Q8	Leucine-rich repeat-containing protein 59
Lsm5	P62322	U6 snRNA-associated Sm-like protein

Luc7l	Q9CYI4	Putative RNA-binding protein Luc7-like 1
Luc7l2	Q7TNC4	Putative RNA-binding protein Luc7-like 2
Lypla2	Q9WTL7	Acyl-protein thioesterase 2
Macf1	B1ARU4	Microtubule-actin cross-linking factor 1
Mak16	Q8BGS0	Protein MAK16 homolog
Map4	P27546	Microtubule-associated protein 4
Mapre1	Q61166	Microtubule-associated protein RP/EB family member 1
Mtdh	Q80WJ7	Protein LYRIC
Mtpn	P62774	Myotrophin
Nat10	Q8K224	RNA cytidine acetyltransferase
Ndufs6	P52503	NADH dehydrogenase [ubiquinone] iron-sulfur protein 6
Ndufs7	Q9DC70	NADH dehydrogenase [ubiquinone] iron-sulfur protein 7
Neb	E9Q1W3	Nebulin
Nedd8	P29595	Neural precursor cell expressed developmentally down-regulated protein 8
Nipsnap1	O55125	Protein NipSnap homolog 1
Nras	P08556	GTPase NRas
Nucb1	Q02819	Nucleobindin-1
Nudc	O35685	Nuclear migration protein
Ogt	Q8CGY8	UDP-N-acetylglucosamine--peptide N-acetylglucosaminyltransferase
Ola1	Q9CZ30	Obg-like ATPase 1
Pabpn1	Q8CCS6	Polyadenylate-binding protein 2
Pafah1b2	Q61206	Platelet-activating factor acetylhydrolase IB subunit beta
Plg	P20918	Plasminogen
Ppme1	Q8BVQ5	Protein phosphatase methylesterase 1
Ppp1ca	P62137	Serine/threonine-protein phosphatase PP1-alpha catalytic subunit
Ppp1r12a	Q9DBR7	Protein phosphatase 1 regulatory subunit 12A
Prpf19	Q99KP6	Pre-mRNA-processing factor 19
Prrc1	Q3UPH1	Proline-rich and coiled-coil-containing protein 1
Psmc3	O88685	26S proteasome regulatory subunit 6A
Psmd13	Q9WVJ2	26S proteasome non-ATPase regulatory subunit 13
Psmd7	P26516	26S proteasome non-ATPase regulatory subunit 7
Psmd9	Q9CR00	26S proteasome non-ATPase regulatory subunit 9
Pspc1	Q8R326	Paraspeckle component 1
Ptms	Q9D0J8	Parathymosin
Rab21	P35282	Ras-related protein Rab-21
Rab5c	P35278	Ras-related protein Rab-5C
Rbm17	Q8JZX4	RNA-binding motif protein 17
Rbm25	S4R2B0	RNA-binding protein 25
Rpl15	Q9CZM2	60S ribosomal protein L15
Rps27	Q6ZWU9	40S ribosomal protein S27
Rtn4	Q99P72	Reticulon-4
Ruvbl2	Q9WTM5	RuvB-like 2
Scp2	P32020	Non-specific lipid-transfer protein
Sec22b	O08547	Vesicle-trafficking protein SEC22b
Sec61g	P60060	Protein transport protein Sec61 subunit gamma
Serpinf1	P97298	Pigment epithelium-derived factor
Serpinh1	P19324	Serpin H1
Sf3b1	Q99NB9	Splicing factor 3B subunit 1

Sf3b2	Q3UJB0	Splicing factor 3b, subunit 2
Sh3glb2	Q8R3V5	Endophilin-B2
Slc3a2	P10852	4F2 cell-surface antigen heavy chain
Smu1	Q3UKJ7	WD40 repeat-containing protein SMU1
Snrnp200	Q6P4T2	U5 small nuclear ribonucleoprotein 200 kDa helicase
Snrnp70	Q62376	U1 small nuclear ribonucleoprotein 70 kDa
Snx2	Q9CWK8	Sorting nexin-2
Srsf4	Q8VE97	Serine/arginine-rich splicing factor 4
Ssfa2	Q922B9	Sperm-specific antigen 2 homolog
Stim1	P70302	Stromal interaction molecule 1
Stk25	Q9Z2W1	Serine/threonine-protein kinase 25
Stmn1	P54227	Stathmin
Sugct	Q7TNE1	Succinate--hydroxymethylglutarate CoA-transferase
Supt16h	Q920B9	FACT complex subunit SPT16
Tars	Q9D0R2	Threonine--tRNA ligase, cytoplasmic
Thoc7	Q7TMY4	THO complex subunit 7 homolog
Tjp2	Q9Z0U1	Tight junction protein ZO-2
Tkfc	Q8VC30	Triokinase/FMN cyclase
Tnks1bp1	P58871	182 kDa tankyrase-1-binding protein
Tomm70	Q9CZW5	Mitochondrial import receptor subunit TOM70
Top2b	Q64511	DNA topoisomerase 2-beta
Trappc3	O55013	Trafficking protein particle complex subunit 3
Twf1	Q91YR1	Twinfilin-1
U2af1l4	Q8BGJ9	Splicing factor U2AF 26 kDa subunit
Ube2g1	P62254	Ubiquitin-conjugating enzyme E2 G1
Ubqln1	Q8R317	Ubiquilin-1
Ufc1	Q9CR09	Ubiquitin-fold modifier-conjugating enzyme 1
Ufd1	P70362	Ubiquitin recognition factor in ER-associated degradation protein 1
Uso1	Q9Z1Z0	General vesicular transport factor p115
Usp15	Q8R5H1	Ubiquitin carboxyl-terminal hydrolase 15
Vapb	Q9QY76	Vesicle-associated membrane protein-associated protein B
Vps35	Q9EQH3	Vacuolar protein sorting-associated protein 35
Wars	P32921	Tryptophan--tRNA ligase, cytoplasmic
Zcchc6	Q5BLK4	Terminal uridylyltransferase 7

† Grey text: Common contaminant families of proteins [57] not used for clarity in STRING network generation.

Supplementary Table S4. Oligonucleotides used in this study.

Target	Purpose	Sequence
Cyrano-F	qPCR	GAAACATAGGCTGGGACAAT
Cyrano-R	qPCR	TGTTACTGGGCTCTGTTT
GAPDH-F	qPCR	GGCCGCATCTTCTTGTG
GAPDH-R	qPCR	GCCGTGAGTGGAGTCAT
Gata6-F	qPCR	ACCTCAGGGGTAGGGGCAT
Gata6-R	qPCR	GGAGGACAGACTGACACCTATGTA
Pdgfra-F	qPCR	CACGCCAGACTGTGTATAAG
Pdgfra-R	qPCR	GTTTGATGGATGGGAGTTTGA
Nanog-F	qPCR	TCTTCTACCAGTCCCAAA
Nanog-R	qPCR	GCTAGTCTTCAACCACTG
Cyrano-1	CHART	AACCAGGACGCCTCACTGAGCAGTC-biotin-TEG
Cyrano-2	CHART	GGCAGTCTCTGCATCCTAAACCATG-biotin-TEG
Cyrano-3	CHART	TTATCGTATCTTGTTTTTGTCCAGG-biotin-TEG
Sense-1	CHART	GACTGCTCAGTGAGGCGTCCTGGTT-biotin-TEG
Sense-2	CHART	CATGGTTTAGGATGCAGAGACTGCC-biotin-TEG
Sense-3	CHART	CCTGGACAAAAACAAGATACGATAA-biotin-TEG
Scrambled-1	CHART	GCCACCACGCGAATTGAAGCTCCGA-biotin-TEG
Scrambled-2	CHART	ACAGACGCGCATCTATCGTCATCGT-biotin-TEG
Scrambled-3	CHART	ACAGACGCGCATCTATCGTCATCGT-biotin-TEG

SUPPLEMENTARY FIGURE 1

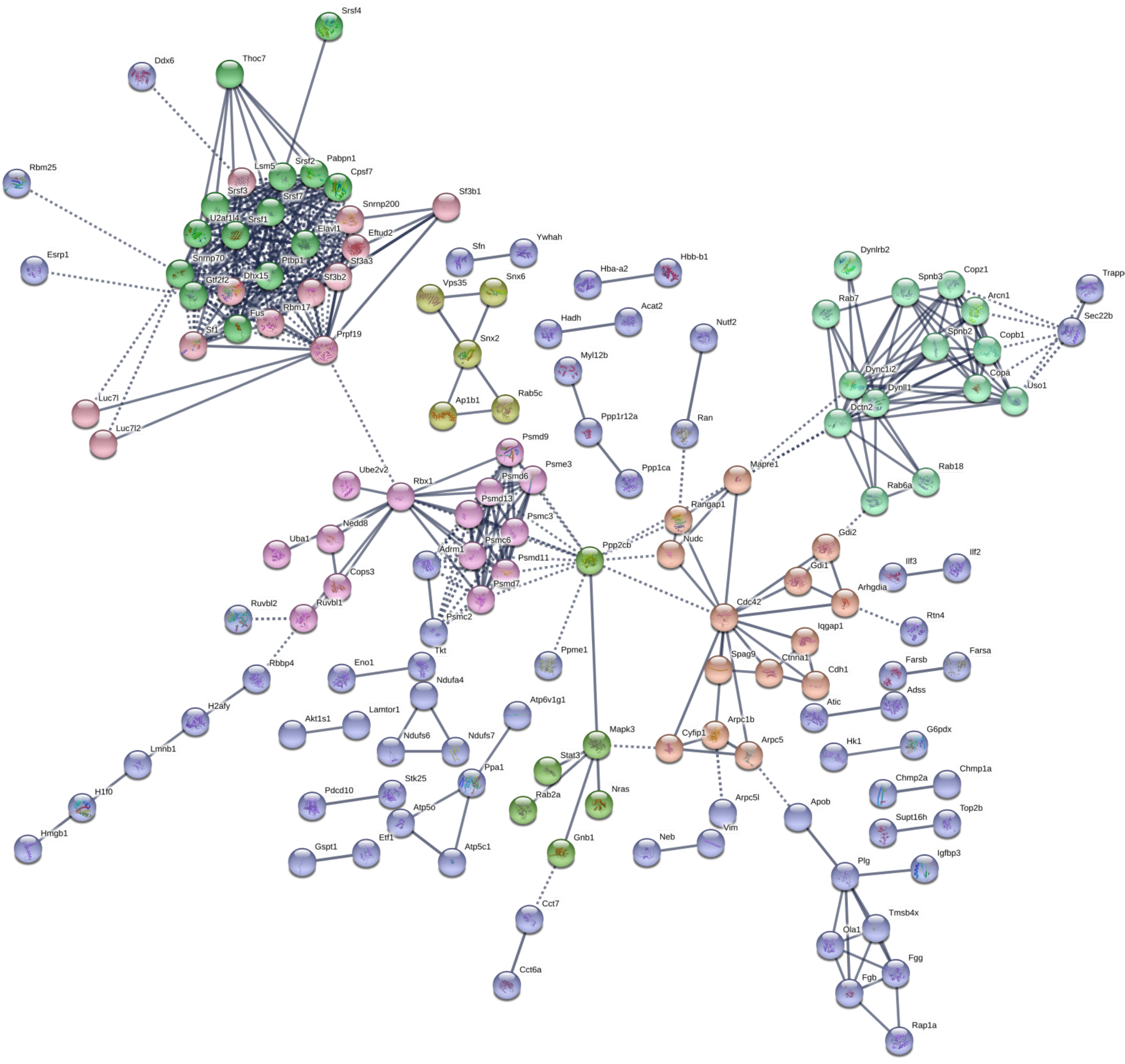

SUPPLEMENTARY FIGURE 2

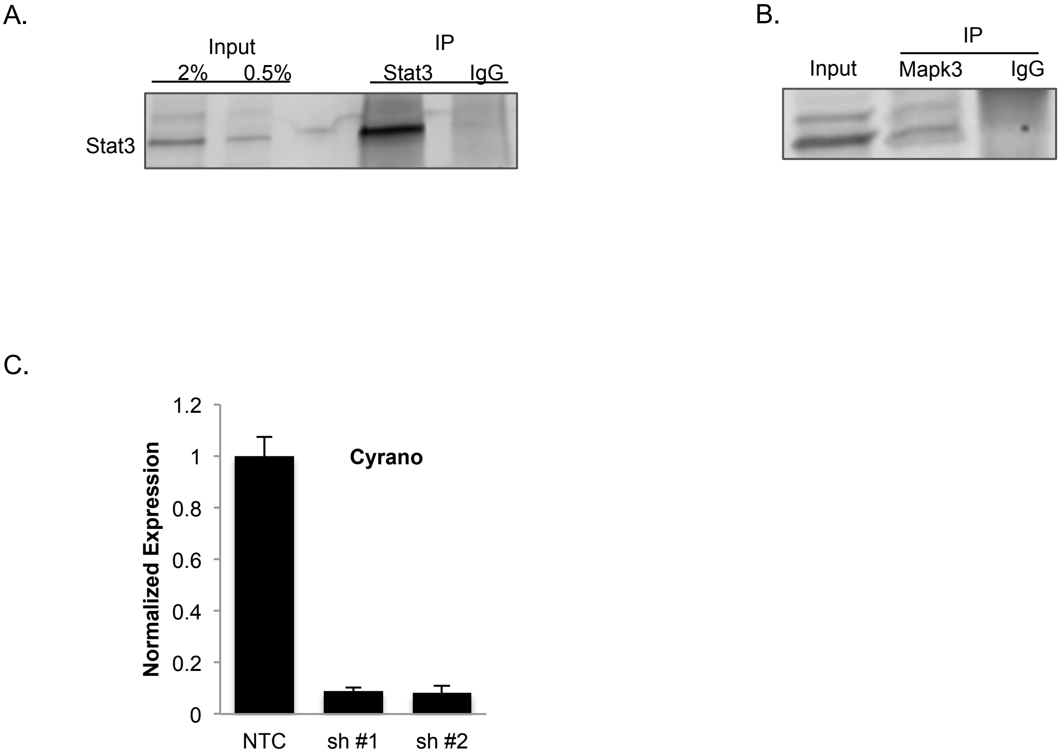

Supplement: Supplementary file 1 — Supplementary Figures and Tables [file 41598_2018_34864_MOESM1_ESM.pdf]
